# Evaluating SARS-CoV-2 Seroconversion Following Relieve of Confinement Measures

**DOI:** 10.3389/fmed.2020.603996

**Published:** 2020-12-18

**Authors:** Juliana Gonçalves, Rita L. Sousa, Maria J. Jacinto, Daniela A. Silva, Filipe Paula, Rute Sousa, Sara Zahedi, Joana Carvalho, M. Guadalupe Cabral, Manuela Costa, Jaime C. Branco, Helena Canhão, José D. Alves, Ana M. Rodrigues, Helena Soares

**Affiliations:** ^1^Human Immunobiology and Pathogenesis Laboratory, Lisbon, Portugal; ^2^CEDOC-Chronic Diseases Research Center, NOVA Medical School | Faculdade de Ciências Médicas, NOVA University of Lisbon, Lisbon, Portugal; ^3^Católica-Lisbon School of Business and Economics, Catholic University of Portugal, Lisbon, Portugal; ^4^Internal Medicine Department IV/Immune Mediated Systemic Diseases (UDIMS), Fernando Fonseca Hospital, Amadora, Portugal; ^5^Immune Response and Vascular Disease Laboratory, Lisbon, Portugal; ^6^Comprehensive Health Research Center (CHRC), NOVA Medical School, Lisbon, Portugal; ^7^EpiDoC Unit, CEDOC, NOVA Medical School, UNL, Lisbon, Portugal; ^8^Computational and Experimental Biology Laboratory, Lisbon, Portugal; ^9^Tissue Repair and Inflammation Laboratory, Lisbon, Portugal; ^10^Rheumatology Department, CHLO, Egas Moniz Hospital, Lisbon, Portugal

**Keywords:** SARS-CoV2, serosurvey, IgA and IgG, pauci/asymptomatic COVID-19 prevalence, post-confinement community setting

## Abstract

Seroprevalence studies are crucial both for estimating the prevalence of SARS-CoV-2 exposure and to provide a measure for the efficiency of the confinement measures. Portuguese universities were closed on March 16th 2020, when Portugal only registered 62 SARS-CoV-2 infection cases per million. We have validated a SARS-CoV-2 ELISA assay to a stabilized full-length spike protein using 216 pre-pandemic and 19 molecularly diagnosed SARS-CoV-2 positive individual's samples. At NOVA University of Lisbon, presential work was partially resumed on May 25th with staggered schedules. From June 15th to 30th, 3–4 weeks after the easing of confinement measures, we screened 1,636 collaborators of NOVA university of Lisbon for the presence of SARS-CoV-2 spike specific IgA and IgG antibodies. We found that spike-specific IgG in 50 of 1,636 participants (3.0%), none of which had anti-spike IgA antibodies. As participants self-reported as asymptomatic or paucisymptomatic, our study also provides a measurement of the prevalence of asymptomatic/paucisymptomatic SARS-CoV-2 infections. Our study suggests that essential workers have a 2-fold increase in viral exposure, when compared to non-essential workers that observed confinement. Additional serological surveys in different population subgroups will paint a broader picture of the effect of the confinement measures in the broader community.

## Introduction

In late 2019, SARS-CoV-2 emerged as a novel human coronavirus, and its subsequent worldwide spread has led to ~47,596,852 infections and to ~1,216,357 deaths (https://covid19.who.int). COVID-19, the clinical disease caused by SARS-CoV-2 infection, spans from mild self-limiting disease to acute respiratory distress and death (1–3). Even though testing capacity has increased sharply in the past months, most of SARS-CoV-2 reported cases have been restricted to symptomatic individuals and those having close contact with confirmed patients. Notwithstanding, subclinical asymptomatic infections are reported to account for ~40–45% of infections and thought to be an important contributor for SARS-CoV-2 transmission (4). Thus, assessing the cumulative prevalence of SARS-CoV-2 infection, including accounting for asymptomatic or subclinical cases, might be critical to underpin the SARS-CoV-2 contagiousness and the success of confinement measures (5, 6).

Serological tests, which detect antibodies specific for SARS-CoV-2, allow for a more accurate estimate of the cumulative prevalence of SARS-CoV-2 infection in a population compared to the viral diagnostic test; as SARS-CoV-2 antibodies, in particular IgG, persist after viral clearance (7). Most of the SARS-CoV-2 serological tests developed so far detect antibodies made against the viral protein Spike (8–13). Spike is a trimeric glycoprotein protruding from SARS-CoV-2 viral membrane that mediates viral entry into the host cell (14, 15). These features make spike the preferential target in the development of serological tests and vaccine candidates. Hence, the serological assay used in this study to characterize anti-SARS-CoV-2 specific antibody response makes use of the trimerized, stabilized ectodomain of the spike protein. It is based on an enzyme-linked immunosorbent assay (ELISA) initially developed in early 2020 by Florian Krammer group at Mount Sinai's CLIA laboratory where it received New York State Department of Health (NYSDOH) and FDA emergency use authorization (8, 9).

On March 2nd 2020, the first case of coronavirus disease 2019 (COVID-19) was diagnosed in Portugal (16). On March 16th confinement measures including the closure of schools and universities and encouragement of remote work were enforced. Even though Portugal has one of the highest SARS-CoV-2 infection testing rate, testing has been mainly limited to symptomatic cases and their close contacts, leaving unaccounted asymptomatic and subclinical cases. Seroprevalence studies provide relevant epidemiological information to determine SARS-CoV-2 viral exposures within individuals and in the population (17). In view of the relative low prevalence of SARS-CoV-2 infection in most of the countries analyzed so far (7, 18–21), ELISA tests being developed need to be not only sensitive but also highly specific (22). Moreover, serological assays must be of straight-forward implementation and provide information that can be easily compared between distinct populations and geographic localizations.

Monitoring the seroprevalence in professional groups that adhered, to distinct extents, to remote work as early as March 8th 2020 is very relevant for evaluating the efficacy of the confinement measures. In NOVA University of Lisbon, even though remote work remained encouraged, the easing up of confinement measures started on May 25th 2020 with collaborators gradually going back to work in staggered schedules. From June 15th to June 30th 2020, 3–4 weeks after easing up of confinement at NOVA University, we performed a serosurvey on 1,636 university's collaborators encompassing seven distinct schools and the rectorate. In this study, we estimated and characterized the antibody profile of a population subgroup that had been under confinement to distinct extents for the 3 months prior to the serosurvey. We found the overall seroprevalence of SARS-CoV-2 spike specific IgG antibodies of the NOVA university community to be 3%. Interestingly, SARS-CoV-2 seroprevalence was 2–4-fold higher in the medical school (6.20%) than in the other schools. This most likely is ascribed to the fact that medical school faculty is composed mainly by clinicians, which as essential workers were precluded from confinement. Curiously, the seroprevalence of the collaborators of the Institute for Tropical Health and Hygiene (4.26%), which functions in articulation with hospitals, was 1.4-fold higher than the overall prevalence. As a corollary of this study, we provide a measure of prevalence of asymptomatic SARS-CoV-2 infections.

## Materials and Methods

### Study Participants and Human Samples

For the setup of our ELISA assay, we used pre-pandemic plasma samples originated from 3 cohorts: 43 samples from healthy donors (HD), 138 samples from rheumatoid arthritis (RA) patients and 35 samples from systemic lupus erythematosus (SLE) patients. All pre-pandemic samples were collected between 2016 and December 2019. In addition, we used serum samples from 19 adult individuals that consulted Hospital Fernando Fonseca between April and May 2020 and were confirmed positive for SARS-CoV-2 by RT-PCR from nasopharyngeal and/or oropharyngeal swabs in a laboratory certified by the Portuguese National Health Authorities. The detailed demographics and clinical characteristics of these 19 SARS-CoV-2 infected individuals are shown in Table 1. Patients were clinically examined, scored for disease severity and classified as: Asymptomatic-committed to the hospital for other complaints, subjected to routine PCR-testing for SARS-CoV-2 but upon medical examination did not exhibit any signs nor symptoms; Mild disease- displayed fever, cough, myalgias, or loss of taste and smell but did not require oxygen supplementation; Moderate disease- required non-invasive oxygen supplementation and hospitalization; Severe disease- required invasive oxygen supplementation and patients were committed to intensive care. NOVA University serum samples (*n* = 1,636) were collected between June 15th and 30th 2020. Serum/plasma were collected by whole blood centrifugation at 1,000 × g for 10 min at room temperature. Plasma/serum was carefully aliquoted and stored at temperature controlled−80°C ultra-low freezer at CEDOC Biobank for subsequent analysis. All samples were heat-inactivated at 56°C for 60 min. All experiments and analyses from human donors were conducted with the approval of local ethics committee, in accordance with the provisions of the Declaration of Helsinki and the Good Clinical Practice guidelines of the International Conference on Harmonization.

**Table 1:** Comparison of demographic and clinical data between asymptomatic, mild and severe group of COVID-19 patients.

**Parameters**	**Asymptomatic group (*n* = 5)**	**Mild group** **(*n* = 13)**	**Severe group** **(*n* = 1)**
Sex			
Females-*n* (%)Males-*n* (%)	2 (40%) 3 (60%)	9 (69.2%)4 (30.8%)	1 (100%) 0
Age categories-*n* (%)			
45–6465+	2 (40%) 3 (60%)	4 (30.8%)9 (69.2%)	0 1 (100%)
SARS-CoV-2 PCR+	100%	100%	100%
PCR+ days to serum collection	19 (11–19.5)	18 (10–24)	24
Comorbidities-*n* (%)			
HypertensionDiabetesHeart FailureCKDCancer	2 (40%) 4 (80%) 1 (20%) 1 (20%) 1 (20%)	5 (38.5%)9 (69.2%)3 (23.1%)2 (15.4%)2 (15.4%)	1 (100%) 1 (100%) 0 0 0
Bacterial Infection-*n* (%)	3 (60%)	4 (30.8%)	1 (100%)
CRP (mg/dL)	2.41 (0.78–5.38)	7.24 (2.63–12.61)^#^	0.41
Ferritin (ng/mL)	711 (346.5–1300)	539 (243–1126)^*^	330
D-dimer (ng/mL)	1864 (1507–10240)	2017 (784–11452)^*^	822
Leukocyte (10^9^/L)	4.6 (4.2–8.5)	8 (4.5–8.95)	22.3
Lymphocyte (10^9^/L)	1.4 (0.9–1.8)	1.3 (0.8–1.5)	8.9
Procalcitonin (ng/mL)	0.11 (0.03–0.28)^#^	0.3 (0.13–0.67)^#^	0.06

* Clinical data missing for two donors. Median calculated out of total available data.

# Clinical data missing for one donor. Median calculated out of total available data.

*CKC, chronic kidney disease; CRP, C-reactive protein*.

### Production of Trimeric Spike Protein

The construct encoding the trimeric pre-fusion stabilized ectodomain of SARS-CoV2 spike (S) protein was kindly donated by Dr Florian Krammer, Icahn School of Medicine at Mount Sinai, New York, USA. Recombinant trimeric spike protein was produced and purified at Instituto de Biologia Experimental e Tecnológica (IBET), Oeiras, Portugal under de Serology4COVID consortium, as previously described (8, 9). In short, His-tagged trimeric spike was produced by transient transfection of Expi293FTM cells (Thermo Fisher Scientific) with a spike plasmid suitable for mammalian cell expression (pCAGGS). All subsequent purification steps were carried out at 4°C. Three days post-transfection, supernatants were collected filtered through Sartopore MidiCaps, concentrated and dialysed with binding buffer by tangential flow filtration, using 30 kDa membranes. The dialysed and concentrated sample was filtered through 0.22 μm membrane and loaded into HisTrap HP columns, equilibrated with binding buffer. Spike protein was eluted with a linear gradient up to 500 mM Imidazole. Fractions containing Spike were concentrated to 1–2 mg/mL using Vivaflow 200 crossflow devices. Removal of imidazole and exchange to PBS buffer was performed by diafiltration with 10 volumes of PBS. Protein concentration was determined by absorbance at 280 nm combined with the specific extinction coefficient. The concentrated and formulated products are filtered through 0.22 μm membrane, aliquoted, snap frozen in liquid nitrogen and stored at −80°C.

### ELISA Assay

A modified in-house ELISA was performed based on the published protocol (8). Coating conditions were assayed by antigen dilution with sera collected from 12 COVID-19 patients and eight pre-pandemic samples collected between 2016 and December 2019. Ninety six-well plates (Nunc, M9410) were used to test coating concentrations ranging from 0.5 to 2 μg/mL. Sera/plasma were heat-inactivated at 56°C for 1-h prior being loaded in the in-house ELISA. High-binding 384-well ELISA plates (Nunc, 735-0114) were coated with spike protein at 0.5 μg/mL overnight at 4°C. After washing three times with 0.1% PBS/Tween20 (PBST) using an automatic plate washer (ThermoScientific), plates were blocked with 3% milk in 0.05% PBST for 1 h at room temperature. Wells were emptied and samples diluted 1:50 in 1% milk powder PBST were added and incubated for 1 h at room temperature. Calibrators included sera from PCR-tested SARS-CoV-2 infected individuals classified in three groups according to their antibody titers: high-, moderate- and low-antibody producers. Three individual samples from each group were used. Negative controls included two pre-pandemic samples and two blank wells. Following PBST washes, secondary antibody was added at 1:25,000 dilution and incubated for 30 min at room temperature. IgA and IgG were detected using Goat anti-Human IgA/IgG-HRP (abcam, ab97225/ab97215). Plates were washed with PBST and 25 μL of TMB substrate (Biolgend, 421101) was added to the wells for ~7 min. The reaction was stopped by adding 12.5 μL of 1 M phosphoric acid (Sigma, P5811) and read at 450 nm on a plate reader (BioTek). Two-hundred and sixteen pre-pandemic plasma samples were used to establish the assay cut-off value for seropositivity at 0.3987 for IgG. The cut-off value resulted from the mean of OD_450_, values from all negative controls plus 3 times the standard deviation. ROC curve analysis determined a 99.53% specificity (with a CI_95%_ of 97.41–99.99%) and 94.74% sensitivity (with a CI_95%_ of 73.97–99.87%) at this cut-off for the serum dilution (1:50) used. The cut-off of 0.3987 was applied uniformly to all assays and the seropositivity was defined as any individual whose OD value lies above the cut-off in serum diluted at 1:50. For IgA the assay cut-off value for seropositivity was calculated in the similar manner at 0.459. ROC curve analysis determined a sensitivity of 57.89% (with a CI_95%_ of 33.50–79.75%) and a specificity of 99.53% (with a CI_95%_ of 97.39–99.99%), at serum dilution 1:50. Inter- and intra-assay coefficient of variability were found to be 5.3 and 2.7%, respectively. Each plate contained 16 calibrators samples from three high-, three medium-, and three low- antibody producers and pools from all high-, medium- and low- antibody producers, two pre-pandemic samples and two blank wells. Endpoint titers were established using a 3-fold dilution series starting at 1:50 and ending at 1:10,9350 and defined as the last dilution before the signal dropped below OD_450_ of 0.15.

### Statistical Analysis

Statistical analysis was performed using GraphPad Prism (version 8.4.2). The non-parametric Kruskal-Wallis test was used to compare the geometric ratios between groups. Spearman correlation test was used in correlation analysis. The non-parametric Man-Whitney test and Wilcoxon test were used as described in figure legends.

## Results

### SARS-CoV-2 Spike Protein ELISA Assay Setup

Our serological assay uses the trimeric ectodomain of SARS-CoV-2 spike protein as antigen and consists in a modified ELISA protocol developed by Florian Krammer group at Mount Sinai's CLIA laboratory where it received New York State Department of Health (NYSDOH) and FDA emergency use authorization (8, 9). To set-up our ELISA assay we made use of serum from 19 individuals consulted at Hospital Fernando Fonseca that were diagnosed positive for SARS-CoV-2 infection by RT-PCR of nasopharyngeal and/or oropharyngeal swabs in a laboratory certified by the Portuguese National Health Authorities. SARS-CoV-2 infection may be asymptomatic or occur associated to varied clinical manifestations including fever, asthenia, myalgia, eventually progressing toward pneumonia and acute respiratory distress (23, 24). Several comorbidities, such as cancer, diabetes and cardiovascular disease may negatively influence COVID-19 severity and mortality (25). Demographics, reason for medical admission (especially important in the case of asymptomatic cases), and relevant co-morbidities for the 19 SARS-CoV-2 RT-PCR positive individuals are contained in Table 1 together with the results of PCR testing. When designing our assay setup, we took care that our sampling of SARS-CoV-2-positive individuals would reflect as well as possible the expected profile found in the NOVA community in which this assay would be run, i.e., a population with mainly asymptomatic or paucisymptomatic disease. To this end, we focus on SARS-CoV-2-positive group in individuals undergoing routine visits to the hospital (e.g., child delivery, oncology, cardiovascular or diabetes consults). Since this population consults were not related with COVID-19, they were composed almost exclusively (18 out of 19) of asymptomatic or paucysimptomatic individuals, which reflects more closely the general population.

**Figure 1:**
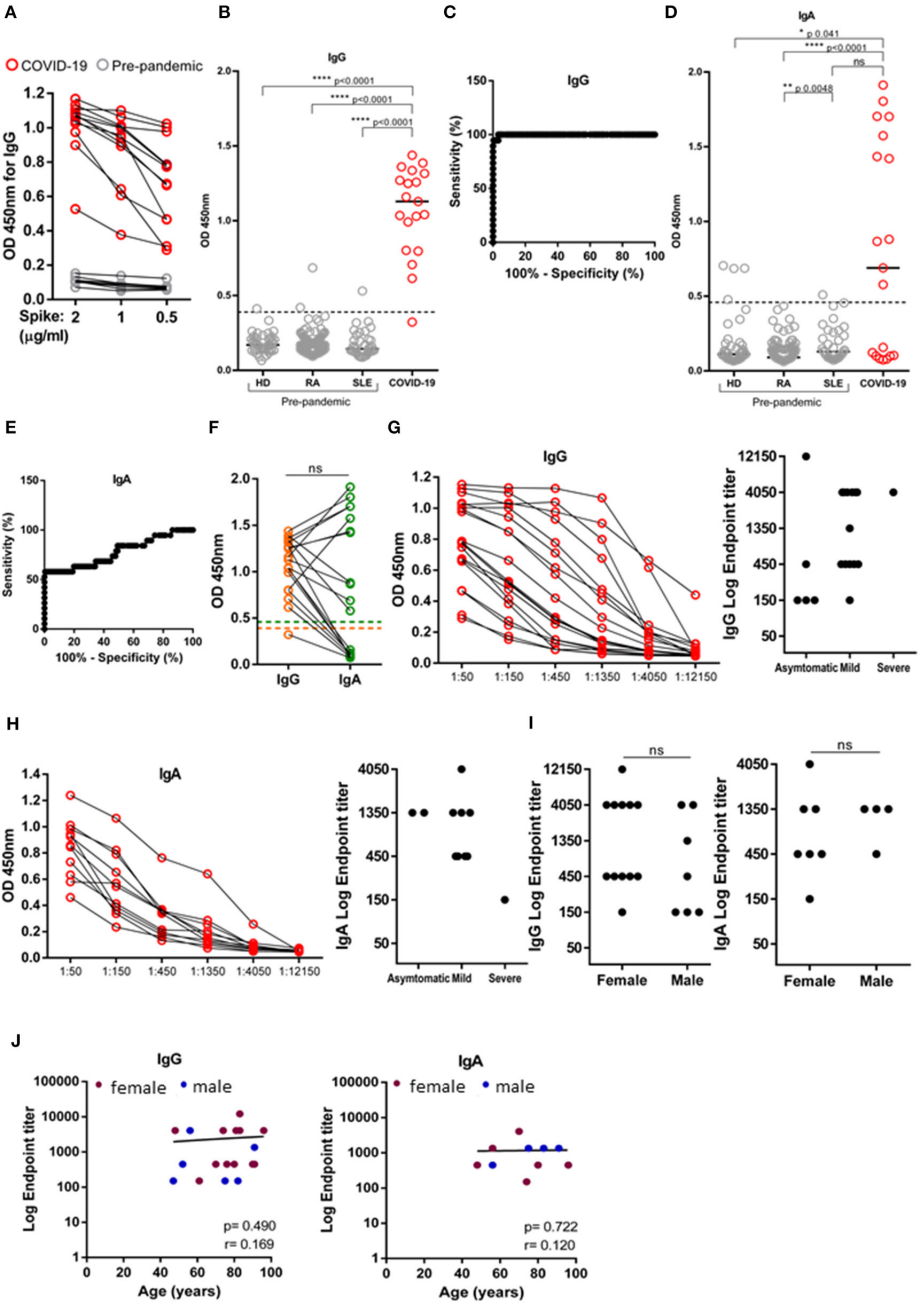
Validation of SARS-CoV-2 ELISA assay. Levels IgG and IgA against trimeric spike protein of SARS-CoV-2 measured in the serum of SARS-CoV-2 PCR-positive individuals (*n* = 19) and pre-pandemic donors (*n* = 216) by absorbance at 450 nm (OD_450_). **(A)** Titration of trimeric Spike protein coating at 2, 1 and 0.5 μg/mL by measuring IgG OD_450_ of 12 SARS-CoV-2 PCR-positive individuals (red) and eight pre-pandemic controls diluted at 1:50 (gray). **(B)** Comparison of IgG levels between 19 SARS-CoV-2 PCR-positive individuals (red) and three pandemic cohorts: healthy donors (HD, *n* = 43), rheumatoid arthritis (RA, *n* = 138) and systemic lupus erythematosus (SLE, *n* = 35), diluted at 1:50. Dashed line indicates test cut off. **(C)** ROC analysis plotting specificity against sensitivity of samples as in **(B)**. **(D)** Comparison of IgA levels between 19 SARS-CoV-2 PCR-positive individuals (red) and three pandemic cohorts: healthy donors (HD, *n* = 43), rheumatoid arthritis (RA, *n* = 138) and systemic lupus erythematosus (SLE, *n* = 35), diluted at 1:50. Dashed line indicates seropositivity threshold. **(E)** ROC analysis plotting specificity against sensitivity of samples as in **(D)**. **(F)** Donor matched OD_450_ values for IgG and IgA of serum SARS-CoV-2 PCR-positive individuals diluted at 1:50. Dashed line indicate, orange: IgG seropositivity threshold, green: IgA seropositivity threshold. **(G)** Serial dilution for anti-spike IgG (left) and anti-Spike IgG endpoint titers (right) according to disease classification (severe, mild and asymptomatic). **(H)** Serial dilution for anti-spike IgA (left) and anti-Spike IgA endpoint titers (right) according to disease classification (severe, mild and asymptomatic). **(I)** Anti-Spike IgG and IgA endpoint titers in SARS-CoV-2 PCR-positive individuals segregated by sex. **(J)** Levels of IgG and IgA endpoint titers against age (females-maroon; males-blue). Data show individual sample values. *P*-values ****p < 0.0001, **p < 0.01, *p < 0.05 were determined by Kruskal-Wallis test **(B,D)**, Wilcoxon test **(F)**, Man-Whitney test **(I)** and Spearman test **(J)**.

First, we proceeded to titrate the concentration of trimeric spike ectodomain used to coat ELISA plates, by comparing the OD_450_ of IgG from 19 SARS-CoV-2 positive and eight pre-pandemic samples diluted 1:50, when plates were coated with 2, 1, and 0.5 μg/mL of spike (Figure 1A). Since spike protein at 0.5 μg/mL increased the dispersion of antibody production profiling without functional loss in detection (Figure 1A), we henceforth used the coating dose of 0.5 ug/mL in our serosurvey. To establish the ELISA assay cut-off, we made use of 216 pre-pandemic samples. Our pre-pandemic cohort (Table 2, *n* = 216) was composed by 43 healthy donors (HD) and 183 patients with chronic inflammatory diseases, rheumatoid arthritis (RA, *n* = 138) and systemic lupus erythematosus (SLE, *n* = 35), whose dysregulated antibody increases the probability of displaying cross-reactive antibodies against self and a variety of infections (26). The assay cut-off was established at 0.3987, resulting from the mean of the OD_450_ values from all negative controls plus three times the standard deviation (8, 9). One of the 19 SARS-CoV-2 individuals did not seroconvert and four pre-pandemic samples had OD_450_ values above cut-off (Figure 1B). These four pre-pandemic individuals were later deemed as false positives as the OD_450_ values were under the threshold once the samples were diluted at 1:150. ROC curve analysis determined a 94.74% sensitivity (with a CI_95%_ of 73.97–99.87%) and 99.53% specificity (with a CI_95%_ of 97.41–99.99%) at serum dilution (1:50) used (Figure 1C). As for anti-spike IgA, the cut-off was established at 0.459 and only 11 out of 19 (57.8%) SARS-CoV-2 positive individuals had anti-spike IgA antibodies at the time of the screen (Figure 1D). ROC curve analysis determined a sensitivity of 57.89% (with a CI_95%_ of 33.50–79.75%) and a specificity of 99.53% (with a CI_95%_ of 97.39–99.99%), at serum dilution 1:50 (Figure 1E). We did not detect any association between OD_450_ values of IgG and IgA (Figure 1F).

**Table 2:** Demographic characteristics of the study populations.

	**Pre-pandemic**		
	**HD**	**RA**	**SLE**
*n*	43	138	35
Age-med (interval)	42 (22–67)	62 (29–91)	55 (23–87)
Age categories-*n* (%)			
19–4445–6465+	25 (58.1) 15 (34.9) 3 (7)	15 (10.9)63 (45.6)60 (43.5)	8 (22.9) 20 (57.1) 7 (20)
Females-*n* (%)	42 (97.7)	118 (85.5)	34 (97.1)
Males-*n* (%)	1 (2.3)	20 (14.5)	1 (2.9)

Calculating end-point titers allows to be more exact in the evaluation of the antibody response ensued and to compare antibody production between distinct cohorts. We calculated IgG and IgA endpoint titers of SARS-CoV-2 positive individuals by serial 3-fold dilution and classified end-point titers of 1:150 as low, 1:450 as moderate, and ≥1:1,350 as high antibody producers, as previously done (7). The majority of SARS-CoV-2 positive individuals were either moderate or high IgG/IgA antibody producers (Figures 1G,H), and were similarly distributed in both genders and across age distributions (Figures 1I,J).

This evaluation of titers dispersion by SARS-CoV-2 positive individuals is important for the setup of our assay, since we will use three samples of low-, three samples of medium-, and three samples of high- antibody producers as quality calibrators in each assay run, when testing our cohort. Additional quality control calibrators will include pools of low-, medium-, and high-antibody producers.

### Seroprevalence in NOVA University Community

**Figure 2:**
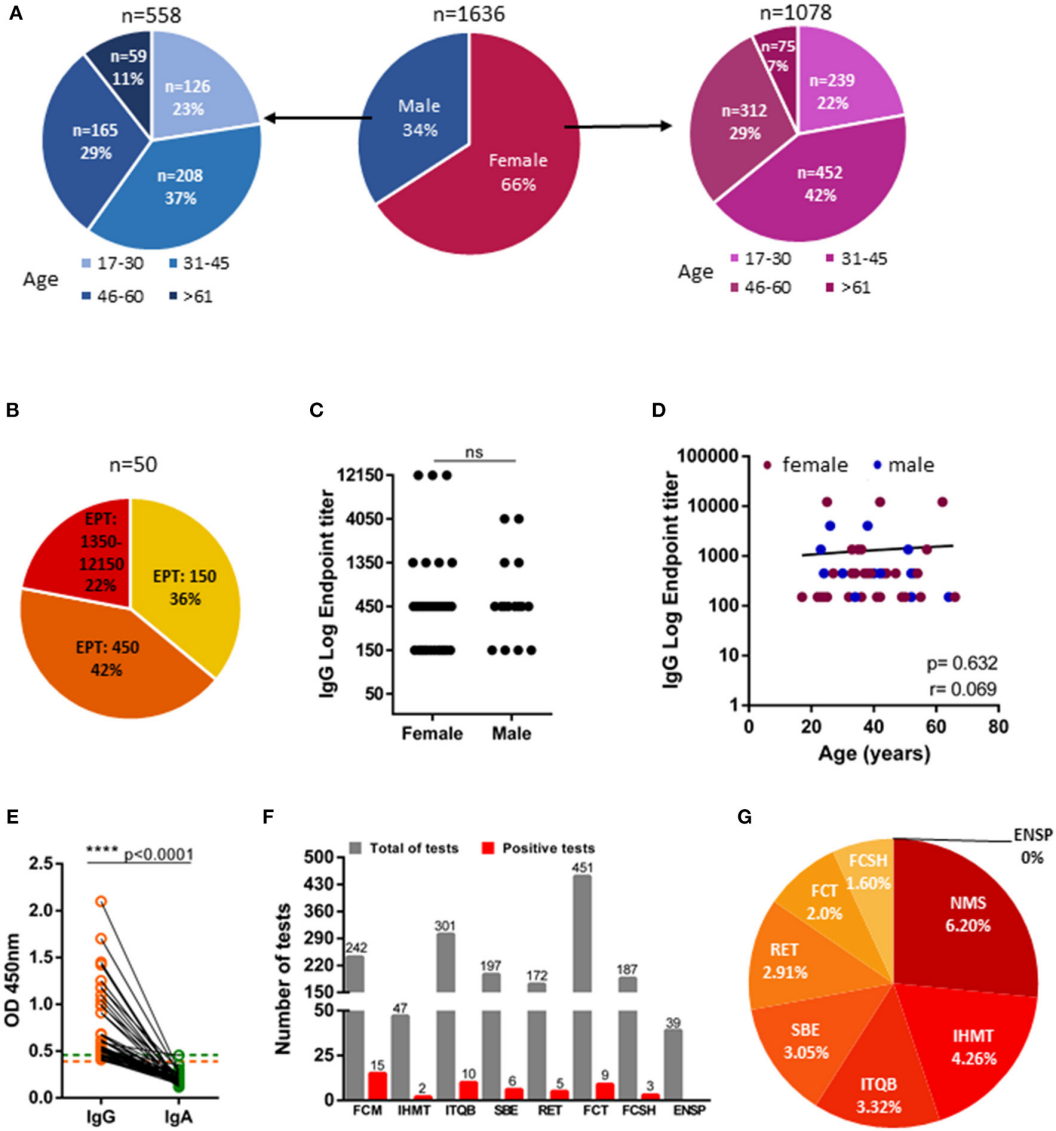
IgG and IgA seroprevalence in NOVA University of Lisbon community. **(A)** Age and gender distribution of NOVA university study cohort (*n* = 1,636). **(B)** Distribution of individuals with IgG titers of 1:150 (low producers), of 1:450 (moderate producers), and ≥1:1,350 (high producers); EPT: Endpoint titer (*n* = 50). **(C)** Anti-Spike IgG endpoint titers in female and male donors (*n* = 50). **(D)** Levels of IgG endpoint titers against age (females-maroon; males-blue; *n* = 50). **(E)** Donor-matched IgA and IgG OD_450_ values of plasma samples diluted at 1:50 (*n* = 50). Dashed line indicate, orange: IgG seropositivity threshold, green: IgA seropositivity threshold. **(F)** Total number of tests performed in NOVA university organic units (gray) and number of positive tests (red) of each unit (NMS: Nova Medical School; IHMT: Institute of Hygiene and Tropical Medicine; ITQB: Institute of Chemical and Biological Technology; SBE: NOVA School of Business and Economics; RET: Rectory; FCT: School of Science and Technology; FCSH: School of Social and Human Sciences; ENSP: National School of Public Health. **(G)** Percentage of positive test considering the total number of tests per organic unit. Data show individual sample values. *P*-values ****p < 0.0001 were determined by Man-Whitney test **(C)**, Spearman test **(D)** and Wilcoxon test **(E)**.

NOVA University of Lisbon collaborators started to ease back to presential work, in staggered schedules, on May 25th 2020. An online registration form was sent to all collaborators of NOVA University of Lisbon and in order to encourage maximum enrollment in the study, sample collection was performed in all the schools and participants were offered a choice of which location was more convenient to them. Two to three weeks later, we collected 1,645 plasma samples from NOVA university collaborators, but demographic data were absent for 9 donors and analysis was restricted to the 1,636 donors that we had available data. Gender distribution was 66% female and 34% male, with ages comprehended between 17 and 76 years (Figure 2A). Study participants self-reported as asymptomatic or paucisymptomatic and had not been diagnosed with COVID-19, with only one having been tested positive by PCR. We found that 50 of 1,636 study participants presented anti-spike IgG. Of those, 22% were high (end-point titers ≥1:1,350), 42% were moderate (end-point titers 1:450), and 30% were low (end-point titers 1:150) antibody producers (Figure 2B). Anti-SARS-CoV-2 IgG antibodies were detected in 34 (68%) women and 16 (32%) men, which reflects the gender distribution of the recruited cohort (66% female and 34% male). In moderate and severe COVID-19 disease women have been found to have higher antibody titers than men (27–30). However, this does not appear to be the case in asymptomatic and mild disease (31, 32). In agreement, our study group, composed overwhelming of asymptomatic individuals, did not provide any differences in IgG end-point titers between females and males (Figure 2C). Only three of the seroconverters were older than 60 years old, consequently, we are unable to draw any conclusion about how antibody titers vary with age in asymptomatic SARS-CoV-2 infections (Figure 2D). Finally, none of IgG seropositive participants had detectable IgA (Figure 2E). The distinct schools complied to the confinement to distinct extents, in view of the specifics of their faculties. To gain some insight into the efficacy of the confinement measures, we reanalyzed our data disaggregated by organic unit: Nova Medical School (NMS); Institute of Hygiene and Tropical Medicine (IHMT); Institute of Chemical and Biological Technology (ITQB); NOVA School of Business and Economics (SBE); Rectory (RET); School of Science and Technology (FCT); School of Social and Human Sciences (FCSH); and National School of Public Health (ENSP). A great extent of Medical School (NMS) faculty consists of practicing clinicians, who, as health care providers, are considered essential workers and exempt from the national decreed confinement. In line with this confinement exemption, we found that anti-SARS-CoV-2 seroprevalence at medical school at 6.20% to be more than 2-fold higher than the average for the university. On the other extreme was the School of Social and Human Sciences (FCSH) that at 1.60% had approximately half of the averaged university seroprevalence (Figures 2F,G).

These data indicate that confinement measures may be efficient at containing SARS-CoV-2 transmission.

## Discussion

Recent data suggest that IgG antibodies specific for SARS-CoV-2 spike protein persist for at least a few months (7, 32). Serosurveys, in particular the ones aimed at detecting anti-spike IgG can potentially estimate the total number of infections, regardless of whether an infection was subclinical at least within this time-frame (7). Here, we setup a serological assay to detect antibodies against spike trimeric ectodomain 3–4 weeks after the easing up of the confinement measures.

In this assay, we used trimeric spike accounting not only the neutralizing antibodies targeting RBD sequence, but also additional epitopes which are suspected to contribute to the overall antibody response (9, 11, 33, 34). To validate the assay, we used serum collected from 19 SARS-CoV-2 positive individuals 11–24 days after RT-PCR testing, a time window when seroconversion has been shown to occur (35–38) and consistent with these previous reports, all but one of the 19 SARS-CoV-2 had seroconverted. Our SARS-CoV-2 positive group encompassed high-, medium-, and low-antibody producers that we then used as internal quality controls to insure the reproducibility and calibration of our assay setup. Importantly, we actively sought that our SARS-CoV-2-positive group would reflect as much as possible the antibody production profile of the general population. In order to do this, rather than recruiting COVID-19 patients, we recruited SARS-CoV-2-positive individuals with overwhelming (18 out of 19) asymptomatic or paucisymptomatic infection. The fact that they were recruited at a major central hospital, was convenient since it ensured that they would not only be screened for SARS-CoV-2, as a routine screening measure, but also that they could be evaluated by clinicians, which could appropriately classify their disease status. We cannot exclude that the reasons that brought the individuals to the hospital, might have affected their antibody response. However, the fact that they were undergoing routine consults plus the advantage of having their SARS-CoV-2 disease status assessed by clinicians, in our opinion, outweighs the possible limitations. Moreover, it would have been logistically difficult to screen asymptomatic individuals outside a health care environment. It is a fact that we have used a limited number of SARS-CoV-2-positive individuals to setup our assays, however the number used is well within the one used in similar studies (3, 11, 31, 32). In addition, variations of this ELISA test developed by Florian Krammer laboratory has been used by multiple groups worldwide. Nonetheless, ideally, SARS-CoV-2-positive individuals should have been recruited from the wide community in a high enough number to ensure the desired performance characteristics of the assay.

In the assay validation we looked if previous conditions that increase the production of antibodies, namely chronic inflammatory diseases such as RA and SLE, could contribute to the assay's background. Even though we detected one RA and one SLE patient with OD_450_ values for IgG well above cutoff, no statistically significant difference was observed when compared to healthy donors. In locations with low prevalence of SARS-CoV-2 infections, serological assays specificity is recommended to be at least 98% (22). Our assay had a sensitivity of 94.74%, specificity of 99.53% with a positive predictive value (PPV) and negative predictive value (NPV) for the found 3% prevalence in the study population of 86.18 and 99.84%, respectively.

Portuguese government decreed national confinement on March 16th 2020 when the official number of SARS-CoV-2 infections reached 331 in a population of ~10 million, with schools closing and remote work enforced when possible. However, it is suspected that the number of cases nation-wide might have been higher than 331, since at the time only symptomatic individuals and those having close contact with confirmed patients were being tested. Serosurveys allow for a more rigorous estimate of COVID-19 prevalence by detecting asymptomatic and paucisymptomatic infections in addition to the symptomatic ones. In this study, we performed serological tests to ~30% of university staff, 3–4 weeks after the easing up of the confinement measures and found a SARS-CoV-2 seroprevalence of 3.0%.

The isotype of the antibody response to SARS-CoV-2 may function as indicators of the type and duration of the immune response. Antibodies of IgA isotype are key to eliminate the virus at the upper respiratory tract mucosal. In COVID-19 disease, IgA antibody production starts early upon infection (~5 days), peaks at 12 day and starkly decreases thereafter (39). As for IgG, it mediates systemic immune responses and it is longer-lasting, with studies pointing that SARS-CoV-2 IgG antibodies could last up until 4 months (7). Notwithstanding, the final duration of SARS-CoV-2 IgA and IgG antibody responses can only be fully characterized in longer, and on-going, longitudinal studies.

The fact that we could only detect IgG, but not IgA, antibodies in NOVA community indicates that in none of the infections took place in the 2–3 week time span between the easing up of the confinement measures and the serosurvey. Moreover, it leaves opens the possibility that the exposure to SARS-CoV-2 might have occurred prior to the enactment of the confinement measures. However, it is impossible to exclude that SARS-CoV-2 exposure might have occurred during confinement.

In our study the participants self-reported as having been asymptomatic or paucisymptomatic in the 3 months prior. Nonetheless, their breadth of antibody production spanned from low to high producers and was in line to the one observed in individuals with clinical signs of COVID-19. Even though a previous report had proposed that asymptomatics possessed lower levels of anti-SARS-CoV-2 IgG antibodies than symptomatic individuals (1), others have observed equivalent IgG production in asymptomatic vs. symptomatic SARS-CoV-2 (21). More studies will be needed to fully characterize antibody production by different SARS-CoV-2 presentations.

Previous studies have shown that the immune response to infection is different in women than in men (40, 41). In the case of hospitalized COVID-19 patients, recent studies have shown that women mount a different immune response, produce higher antibody titers and have less mortality (27, 28). However, the association between gender and antibody titers appears to be less clear in mild or asymptomatic SARS-CoV-2 infection with men and women producing similar antibody titers (31, 32). Similarly, in our cohort of asymptomatic or paucisymptomatic individuals we did not detect significant differences in antibody titers between men and women.

The assessed seroprevalence of 3% is low, nonetheless it's in alignment with the seroprevalence observed in national surveys (18, 19), as well as surveys of local communities (42). Moreover, it is in line with the national survey results involving 2,100 participants and performed in the same post-confinement time window, which found a seroprevalence of 2.9% (https://www.publico.pt/2020/07/31/ciencia/noticia/inquerito-serologico-44-pessoas-anticorpos-sintomas-covid19-1926595). Since confinement measures were implemented national wide, with only exceptions for essential workers, in order to have an idea of the efficacy of the confinement measures we broke down our analysis by school. The medical school stood out, as its faculty is mainly composed of practicing clinicians, who, as essential workers could not comply to the confinement measures. Medical school seroprevalence was, at 6.20%, more than 2-fold of the university average and it was ~4-fold higher than the one observed at the School of Social and Human Sciences (FCSH). The seropositive cases identified at medical school were not linked COVID-19 patient care. Moreover, Portuguese hospitals implemented routine RT-PCR testing and adopted personal protective equipment, measures shown to dampen the risk of transmission in a clinical setting (31, 43). Even though, it is possible that, at least part, of higher seroprevalence in medical school might be due to viral transmission in a social activities (meals, transportation, etc.), we are unable to completely extricate the contribution of non-compliance to confinement from the higher exposure risk in a hospital setting.

Our study has other limitations, as our population sampling is biased and it does not reflect the entire population, namely children and elderly are absent. Our participation rate (~30%), might both enrich for people that suspected having had contacts or mild symptoms, or, on the contrary, be under representative of people with higher risk of infection.

In conclusion, our study suggests that confinement measures might have played a mitigative effect in SARS-CoV-2 transmission and that essential workers have a 2-fold increase in viral exposure, when compared to non-essential workers that observed confinement. Additional serological surveys in different population subgroups encompassing distinct types of essential workers will paint a broader picture of the effect of the confinement measures in the broader community.

## Data Availability Statement

The raw data supporting the conclusions of this article will be made available by the authors, without undue reservation.

## Ethics Statement

The studies involving human participants were reviewed and approved by Ethics Committee of Nova Medical School and Hospital Egas Moniz. Written informed consent to participate in this study was provided by the participants' legal guardian/next of kin.

## Author Contributions

JG and HS customized ELISA assay and analyzed the data. JG, RLS, DAS, SZ, and HS acquired and processed blood specimens. JG, RLS, and MJJ carried out ELISA assays. MGC participated in initial ELISA assays. JDA, FP, JC, AMR, HC, RS, MC, and JCB coordinated sample acquisition. AMR and HC designed the epidemiological survey. RS carried out epidemiological survey. HS conceived the project, supervised the study, interpreted the data, and wrote the manuscript. All authors commented the manuscript.

## Conflict of Interest

The authors declare that the research was conducted in the absence of any commercial or financial relationships that could be construed as a potential conflict of interest.
